# The Panoramic View of Ecuadorian Soil Nutrients (Deficit/Toxicity) from Different Climatic Regions and Their Possible Influence on the Metabolism of Important Crops

**DOI:** 10.3390/toxics11020123

**Published:** 2023-01-26

**Authors:** Raluca A. Mihai, Erly J. Melo Heras, Vanessa A. Terán Maza, Iván A. Espinoza Caiza, Eliza A. Pinto Valdiviezo, Rodica D. Catana

**Affiliations:** 1 CICTE, Department of Life Science and Agriculture, Universidad De Las Fuerzas Armadas—ESPE, Av. General Rumiñahui s/n y, Sangolquí 171103, Ecuador; 2 Institute of Biology Bucharest, Romanian Academy, 296 Splaiul Independentei, 060031 Bucharest, Romania

**Keywords:** agricultural sector, climate, crops of commercial interest, plant defense systems, secondary metabolites, soil characteristics, toxicity

## Abstract

Soil nutrients influence all stages (reproduction, growth, and development) of a plant species’ life, and it is known that the deficit and/or toxicity of one or more nutrients has negative effects on the production of crops of commercial interest. Ecuador represents one of the “mega-diverse” countries in the world, with an agricultural sector of great importance, due to its contribution to the country’s economy. This review provides a panoramic view of soil nutrients from different climatic regions of Ecuador and revises the importance of knowledge about the possible influence of nutrients from the soil on the plant metabolism able to influence the crop resistance against pathogens or to enrich the biological characteristics of these crops.

## 1. Introduction

Ecuador is located in the Northwest of South America (01°28′ N; 05°01′ S and 75°11′ E to 81°01′ W) with borders represented by the Pacific Ocean, Colombia, and Peru. The country is crossed by the Andes Mountains, with elevations from 0–6.300 m.a.s.l. (the snow-capped Chimborazo), and with intense tectonic and volcanic activity [1]. With a total area of 277 thousand km^2^ and about 18.2 million inhabitants, Ecuador has a privileged location on Earth being located in the tropical belt, just above the equinoctial line. Its territories receive luminosity and insolation with light twelve hours a day throughout the year.

Continental Ecuador is divided into three regions (Coastal, Andean, and Amazonian areas), clearly differentiated from each other through topography, climate, and vegetation [2]. The Coastal area is a region of the greatest agricultural expansion and economic dynamism, with great commercial, maritime, and aquaculture activity. The Andean region, with fertile inter-Andean valleys and slopes, constitutes the areas with the highest population density and the greatest pressure on natural resources, especially soil and water. The Amazonian region is a zone with conflicting options between the productive and the environment, being also an oil-producing region.

Despite its limited size, Ecuador has 24 bioclimatic formations or vegetation systems and a high biodiversity, which are suitable to develop different agricultural activities that allow products to be obtained according to market demands. In Ecuador, 29.8% are mountains and forests, 29% are cultivated pastures, while natural pastures represent 12.84%, the moors 4.59%, and land cover by other species 1.65%. Permanent crops are located in 11.83% of Ecuadorian soils.

The agricultural sector is of great importance because it is one of the sectors that contributes most to the country’s economy (overcoming social problems such as poverty) and is a source of foreign exchange through exports [3,4].

## 2. The Climate in Ecuador

The Ecuadorian climate is varied, being greatly influenced by the topography, with significant changes over short distances due to the influence of several factors such as altitudinal gradient, the direction of the mountain ranges, distance from the Pacific Ocean, and ocean currents, as well as winds. Of all the mentioned factors, the one with the greatest influence on climate is altitude [5].

Due to their productive nature, volcanic soils are a significant resource for agriculture; in Ecuador, 31% of the territory has volcanic soils associated with the Cotopaxi, Tungurahua, Sangay, and Reventador volcanoes [5].

The Ecuadorian altitude generates a wide temperature gradient, with an annual average of 0–26 °C. There is a very close relationship between elevation and temperature. However, the Amazonian area has a higher temperature than expected from its elevation while the opposite happens in the dry scrub of the Coast. Altitude influences the amount of rainfall because cold air has little ability to retain moisture. Therefore, the highlands have low rainfall, while the lowlands (up to 2000 m elevation) show wide variation in the amount of rain received throughout the year. Generally speaking, the paramos (tundra ecosystems) receive little rainfall (less than 1.500 mm per year) compared to Andean and lowland forests. The regions that receive the highest levels of precipitation are the western piedmont forest of the northern Andes and the lowland forest of Chocó [6].

Thanks to the climatic conditions, Ecuador has developed agricultural activities that allow it to obtain products according to market demands; the most representative within the country’s economy is the cultivation of bananas, cocoa, African Palm, coffee, and sugarcane, among others [4,7]. According to Portilla, 2018 [2], the characteristics of climates per Ecuadorian regions are represented by:

- Coastal Climate. This region has a tropical or equatorial climate, whose average annual temperature varies between 22 and 26 °C. It is characterized by constant rainfall unevenly in different places and throughout the year; the main rainy months are between December and mid-May, a period considered winter. This inequality in rainfall is due to the effect of the marine currents of Humboldt and El Niño. Mainly, two major climatic zones of the Ecuadorian Coast are considered: hot-cool-dry and hot-hot-humid;
- Andean Climate. The climate of the Andean region is very varied, due to the presence of the Andes Mountain range and the winds that blow through the valleys and plains. The following climates, known as climatic floors or steps, are located in this region: tropical Andean, subtropical Andean, temperate, cold, and glacial;
- Amazonian Climate. In this region, the climate is the same as that of the internal coast—hot, hot-humid. The temperature varies between 22 and 26 °C. This region is the wettest region of the country being an area subject to abundant precipitation (>3.000 mm/year). The flanks of the Andes form a densely cloudy area because large masses of steam from the Atlantic and the Amazon jungle condense.

## 3. Agricultural Soils of the Ecuadorian Regions

The agricultural production of Ecuador is rich and varied thanks to the fertility provided by the soil. Some areas with different soil types are suitable for crops or other species without agronomical interest. In some regions of the country, there are infertile soils due to the conditions of their existence such as volcanic formations, rocky soils, and presence of toxic elements that prevent the normal development of plant species. Moreover, there are regions rich in nutrients suitable for flora and agricultural plantations. The climatic conditions generated by the geographical location (on the equator), the ocean currents, and the influence of the Andes mountain range contribute to great developments and stability that favor the agricultural sectors of the country [8].

In Ecuador, the soils have been classified at the level of Order according to Calvache-Ulloa, 2022 [9]. The soil orders and some characteristics are presented in Table 1.

### 3.1. Soil Characteristics of the Coastal Region

**Figure 1:**
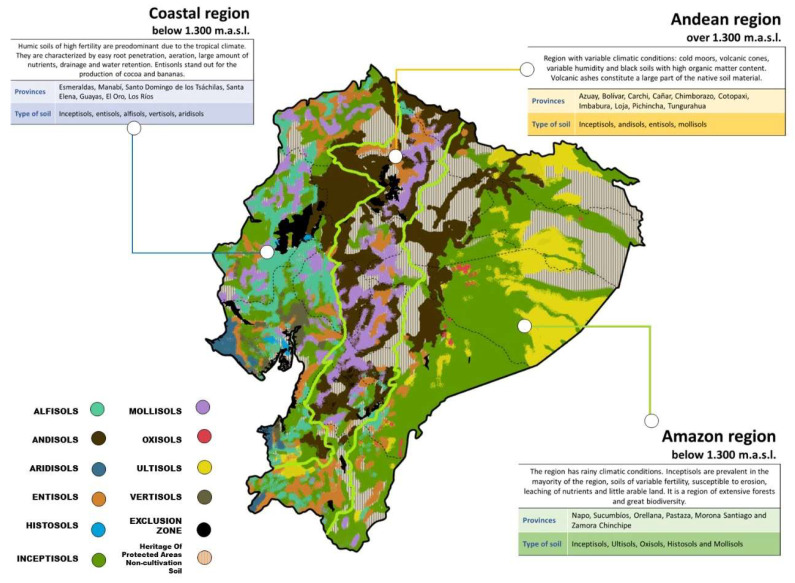
A comprehensive map of the soil characteristics of different Ecuadorian regions.

The soils of the coastal zone contain humic nutrients that provide the growth of crops. Due to the influence of the tropical climates, the Coastal region is beneficial for the development of agricultural products. These soils assure a high agricultural production, which is linked to the economic income of the country and with the commercialization of products (bananas, cocoa, *African Palm*, coffee, etc). The Coastal region assures the food security of the regions of Ecuador as well as to countries where several products are exported [8]. The physical characteristics of the soil of the entire coastal region allow its structure to be known and depending on this, which type of crops should be planted. The characteristic soils of this region are: vertisols, alfisols, entisols, aridisols, mollisols andisols, and inceptisols (Figure 1). Soil structure is characterized by its texture and organic matter content. Depending on their structure, the soils from this region are represented by sand, silt, and clay. This land has the facility for root penetration, aeration, drainage, and water storage capacity and also has large amounts of nutrients. It is characterized by the transport of oxygen and carbon dioxide, giving way to a healthy and quality land operation. The study of the characteristics of the land leads to knowledge of its mineralization and favorable nutrients for agricultural development, and knowing which land is suitable for the various products, whether permanent or transitory. Consequently, the differences in mineral stability will be an important factor in determining the change in mineralogical composition with particle size. These changes are particularly marked as we approach the size of the clay fraction [24]. The entisols (in which cocoa and banana crops are found in the Balao and Naranjal cantons of the Guayas province) occupy an area of 1.324.302 ha, which represents 6% of the mapped national territory, with a large part of them located on steep slopes (>40 to 70%) of the mountainous reliefs. It is worth mentioning that 520.573 ha has an agricultural purpose [14].

### 3.2. Soil Characteristics of the Andean Region

The Andean region includes the areas located over 1300 m.a.s.l. to the peak of the mountains (the snow limit), of both the eastern and western cordillera of the Andes. The lower altitudinal limit of the Sierra lowers gradually towards the south of Ecuador, up to approximately 1000 m.a.s.l. in the province of Loja. It has two mountain chains that run parallel from north to south and enclose intermediate depressions, approximately 40 km. wide, in which valleys are separated by transverse chains called knots. It includes different types of soil: Andisols (potato, cereals), Entisols, (Inceptisols), and Mollisols (legumes, maize, herbs) (Figure 1) [9].

According to Moreno et al., 2022 [25], the characteristics of the Andean region can be synthesized in the following geosystems:

(a) Cold peaks of the Andean Mountain ranges, with inherited landscapes and paramo landscapes with cold or very cold climates and daily night frosts, with volcanic cones of different ages and little evolved black soils with high contents of organic matter. The climate does not favor agricultural activity, but these areas are used for extensive and localized sheep grazing;
(b) External slopes of the Andes, with vigorous and highly dissected modeling on various ancient rocks. The climate is humid to very humid with the presence of heavy cloud cover, conditions that have favored the formation of perennial arboreal zones. The soils are ferralitic with a partial cover of recent volcanic ash and are rejuvenated by erosion;
(c) Inter-Andean basins in the north of the Sierra with volcano-sedimentary fills, where the volcanic morphogenesis promoted by the presence of large recent and active stratovolcanoes is evident. The soils in this area have developed widespread pyroclastic coatings and are subject to intense agricultural activity;
(d) Inter-Andean basins in the center of the Sierra with ancient, volcanic, and metamorphic basements, where there are no volcanoes or recent pyroclastic coatings. The area is fragmented into two sets of parallel sedimentary basins whose coverage is distributed in a topo-climo sequence where the soils of the upper part are ferralitic, often humiferous, those of the middle zone are moderately evolved with mollic or vertic characteristics, and those of the lower zone are little evolved;
(e) Indentations and inter-Andean River valleys with relatively rich alluvial soils in the valleys and on which there is diversified agricultural activity;
(f) Reliefs of the Sierra Austral of ferralitic-fersialitic soils, located in a paleo-topo-climo-edaphological sequence of large valleys and orthogonal depressions where the climatic stratification from humid to arid is clear.

The soil-forming factors are mainly the following: parent material, relief, climates, organisms (flora and fauna), and time. Volcanic ashes constitute, for the most part, the source material of soils, determining certain special characteristics due to the existence of a wide climatic variation. These pyroclastic materials come from the active volcanism of the Quaternary; however, the ashes do not have the same characteristics, since there are layers from different volcanoes and eruption times, determining the existence of differences in the soils. In the Interandean Alley, rainfall is generally related to altitude. Thus, they become drier in the lower part of the basins, and more humid as one ascends the flanks of the mountain range. In the Inter-Andean Alley, the types of reported are Entisols, Vertisols, Inceptisols, Mollisols, Histosols, alfisols, and Andisols [11].

### 3.3. Soil Characteristics of the Amazonian Region

The Ecuadorian Amazon Region represents the largest natural region of Ecuador with approximately 45% of the National territory. Due to its natural forests and extraordinary biodiversity, it constitutes an ecosystem of great local and global interest. From the perspective of sustainable and agroecological management, any future production system which will be developed in the Amazon must be based on uses compatible with the forest, since more than half of the territory (52.7%) has the potential for such use. Extremely rainy weather conditions, with poorly fertile soils that are susceptible to nutrient leaching and erosion, would explain the region’s poor suitability for traditional agricultural activities, but its suitability for productive systems analogous to the forest for conservation systems [26].

The most representative soil orders present in the Amazon region are Inceptisols, Entisols, Histosols, and Mollisols (Figure 1). There are large areas with a pH lower than 5.5, which denotes a limitation for the proper development of crops in this area [27].

## 4. Main Crops of Commercial Interest Cultivated in Ecuador

Since its foundation, Ecuador has based its production on primary resources: cocoa, bananas, coffee, tuna, flowers, shrimp, and others that are obtained directly from nature and whose commercialization does not involve further transformation or added value.

The agricultural sector is of great importance since it is one of the activities that generates the most income, contributing to the generation of employment, and is a fundamental pillar of the national economy [28]. According to the survey of surface and continuous agricultural production (ESPAC) of the year 2021, Ecuador has 12.32 million hectares of land used for permanent crops, of which 47.91% corresponds to forests and woodlands. Of the cultivated area, cocoa employs 41.83%, followed by African palm and banana. Banana and African palm are appreciated as fresh fruit, while cocoa is appreciated as a dried almond [29].

### 4.1. Banana (Musa sp.)

Species belonging to the Musaceae family are perennial crops characterized by a rapid growth rate and are the main staple food in tropical and subtropical countries [30]. With respect to the total value of production in more than 120 developing countries and with >106 million t/year, banana is considered the fourth most important food in the world after rice, wheat, and milk [31]. The main producers are China, India, the Philippines, Indonesia, and Ecuador [32].

In Ecuador, banana *(Musa* AAB variation) is considered a crop of growing socioeconomic importance as the fourth largest producer of this fruit in the world (7.931.000 t/year), besides being the second most exported product after petroleum [33]. Banana represents one of the most important non-oil exports in Ecuador, recognized worldwide for its flavor and excellent quality, making Ecuador a world competitor in the export of this tropical fruit [34]. Bananas are produced on the Coastal region, mainly in the provinces of El Oro (41%), Guayas (34%), and Los Ríos (16%) [7].

Banana is a vital crop susceptible to pathogenic factors and several environmental stresses that negatively affect its growth and yield, such as increases in global temperatures, drought, salinity, pests, and diseases [30].

### 4.2. Cocoa (Theobroma cacao)

*Theobroma cacao* (Malvaceae family) is a perennial plant native to Central and South America. It grows in tropical zones at temperatures between 24 and 26 °C, where the soils are loamy and deep.

Temperature, relative humidity, and rainfall are determining factors in cocoa productivity. The ideal environmental conditions for cocoa cultivation are represented by 15–32 °C, and 1500–3000 mm water. A rainfall below 1500 mm demands additional supplemental irrigation with water, while rainfall above 3000 mm leads to susceptibility to infections caused by microorganisms such as *Phytophthora palmivora* [35]. According to Minimol et al. [36] extremes of hot or cold temperatures influence cocoa flowering, which is consequently reflected in production yields, rainfall promotes flowering, and in the summer season pollen fertility, stigma receptivity, and flowering are lower than in the raining season. The ideal relative humidity (RH) for cocoa is in a range between 70 and 80%, with a higher RHs, susceptibility to pathogens has been seen, and with lower values plant defoliation is induced due to the leaves becoming soft and falling. Another factor is shade, which modifies the microclimate of crops; however, it has been seen that cocoa yields are favored in areas with little shade, where exposure to sunlight is allowed [37].

In Ecuador, cocoa is mainly grown in the coastal region in the provinces of Manabí, Los Ríos, Guayas, and Esmeraldas. In the Sierra, cocoa is also grown in the provinces of Cotopaxi, Bolivar, and Cañar, but with less participation than the coast. The Amazon region also grows this fruit in the provinces of Orellana, Sucumbíos, and Napo; however Zamora Chinchipe has also made its way onto the map of fine aroma cocoa producers [38].

As a raw material, cocoa is classified into two types: *Cacao Fino de Aroma*, *Cacao Nacional or High flavor* and *Cacao CCN51*, “bulk” or “common”. A total of 87% of its production is destined for exports as beans with the United States, Holland, Mexico, Indonesia, Germany, and Belgium as destination countries [38].

### 4.3. African Palm (Elaeis guneensis Jacq.)

The African Palm is a crop that takes 2 to 3 years to bear fruit and is able to produce for 25 years. It is one of the 17 main oils and fats produced worldwide thanks to its profitability, especially when produced in lowlands of tropical regions. In Ecuador, the palm-producing areas are located in the provinces of Esmeraldas, Santo Domingo, Los Ríos, Sucumbíos, and Pichincha, generating two types of oils as products: African palm oil and African palm kernel oil [39]. Palm production is developed in different areas by small producers (in San Lorenzo and Shushufindi) or by large producers (in Esmeraldas and Sucumbíos). In 2021, African palm generates USD 139.3 million and the province of Esmeraldas has the highest production (37.81%) [40].

## 5. Soil Characteristics Necessary for the Optimum Cultivation of Crops

Ecuador is an eminently agricultural country with large amounts of fertile and productive land. Agricultural production can generate a transformation in the quality of the soil that existed before the processes of obtaining food, either due to inadequate management of the systems, lack of technology, or the ecosystem itself.

### 5.1. Banana

Bananas are grown on various types of soils that can be fertile, such as inceptisols with a high potassium storage capacity and andisols with moderate potassium release, and can even be grown in soils with low fertility such as ferrasols and acrisols.

**Figure 2:**
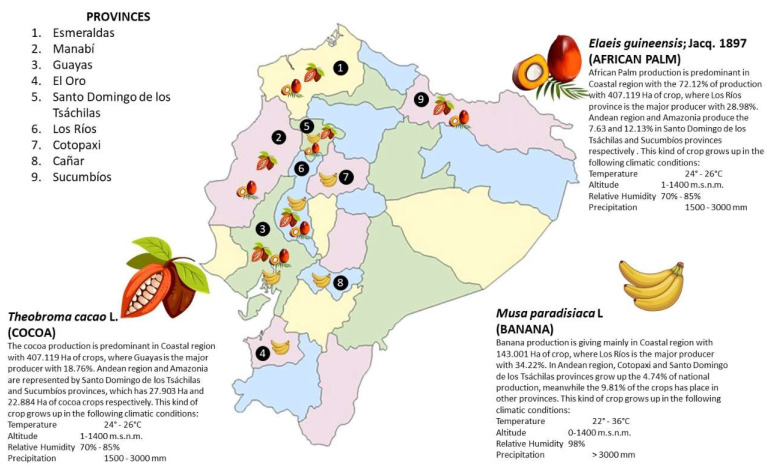
A brief summary of the most important crops in Ecuador.

The conditions for the good development of banana crops are shallow soils, free of loams or clays (>60% clay) and the avoidance of extreme waterlogging (bananas do not tolerate). The best pH range for good banana growth is 5.5–8.0 (Figure 2). A low pH (4.5) reduces the yield by up to 50% due to the low availability of important nutrients such as phosphorus, especially in old tropical soils that fix phosphorus. Nutrient requirements for banana are in the following order: potassium > nitrogen > phosphorus. In addition, it requires significant amounts of calcium and magnesium [41].

### 5.2. Cacao

Cocoa is a crop whose yield is particularly dependent on the level of light (low light, low yields). The environmental conditions that favor cocoa plantations are temperatures between 23 and 25 °C, rainfall between 1200 and 1300 mm/year, and humidity between 70 and 85% (Figure 2). Regarding crop altitude, cocoa is produced in a range from 0 to 1400 m.a.s.l. in areas closer to the equator, requiring soils with a deep and loamy loam to clay loam soils with good water retention, fertile, a percentage of organic matter of at least 3%, and a pH between 6 and 7. Soils that should be avoided for cocoa production are those located on steep slopes or those that are stony, shallow, sandy, near the sea, or very clayey soils. Marshy soils are also not suitable for this type of crop [42].

### 5.3. African Palm

The cultivation of African palm contributes to the generation of jobs and favors the national economy; therefore, the determination of its agroecological conditions is very important for the efficient development of the plant. Oil palm crops are grown on flat land with slight undulations or on gentle slopes, where the soil type is loam, silt loam, clay loam (<35% clay), silty clay loam, sandy clay loam, sandy clay loam, or silty clay loam, without stones or with few rocks. Palm crops are established in an altitudinal range from 0–600 m.a.s.l. at a temperature that can range from 24 to 26 °C, in soils with a slightly acidic (5.6–6.5) or neutral (6.5–7.5) pH, with salinity less than 2 dS/m, in areas with 2400–3000 mm/year, and a medium to high fertility level [42] (Figure 2) [43].

## 6. Nutritional Composition of Agricultural Soils

The soil characteristics are very important to avoid diseases and are essential for the reproduction, growth, and development of the plant species. The characterization of the soil shows the overexploitation of the activities that involve agricultural and livestock activities [9]. Soil fertility depends on the interaction between physical properties (flow of water, air, and nutrients through the pores), chemical properties (pH, cation exchange), and biological properties that directly affect the availability of nutrients to plants [44].

Plants require micro- and macronutrients that are obtained from the soil or through fertilizers and manure. The main processes involved in the release and fixation of nutritional elements in soils include dissolution, precipitation, and adsorption–desorption. Macronutrients required by plants in large quantities are nitrogen (N), phosphorus (P), and potassium (K). Elements such as iron (Fe), manganese (Mn), and zinc (Zn) are required in smaller amounts and are called micronutrients [44]. The plant requires 17 elements to complete its cycle successfully through air and water to obtain essential nutrients such as carbon (C), nitrogen (N), and oxygen (O).

The amount of minerals and nutrients can be influenced by their taxonomy and by the organic matter that will contribute to the fertility status [45]. Human activities with extractive and intensive agricultural practices have generated soil degradation, negatively affecting fertility and productivity; consequently, there are nutrient deficiencies in harvested crops. Nutrient availability may also be limited by natural deficiencies, as nutrients are highly immobilized in the solid phase or soil weathering processes [44].

Knowledge of the agroecological requirements of plants serves as a basis for the proper management and development of food crops. In turn, environmental conditions have a determining effect on crop productivity. Factors such as light, temperature, soil, and nutrients are important aspects of crop management [46]. All factors are correlated, and this relationship can influence the biological traits of the plant. Precipitation can contribute to plant height, while soil nutrients influence leaf size, leaf area, seed size, and respiration rate, among others.

In Table 2 the edaphic characteristics and climatic factors necessary for the main crop development in Ecuador are shown.

## 7. Importance of Nutrients in the Plant Metabolome

The plant metabolome represents the primary (sugars, amino acids, fatty acids, etc.) and secondary metabolites (terpenes, flavonoids, phenolic compounds, etc.), which are direct implicated in the different growth and development processes of the plants. Adequate plant growth requires nutrients, energy, and a good biosynthetic capacity, factors that are influenced by different environmental factors. Minerals are directly involved in plant defense by forming a structural part of different types of molecules. A deficiency or excess of any of the nutrients required by plants for proper development can induce changes in the architecture of these organisms as a strategy to capture as much nutrients as needed [49]. The minerals in the plants’ metabolome is summarized in the Table 3.

Nutrients are considered as the first line of defense against pests by activating enzymes or producing metabolites such as lignin, phytoalexins, and phenols. N, P, and K are the most studied minerals because they are usually not available in crop soils and because they represent several benefits to plants. Minerals such as Mn, Fe, Cu, and Zn, called micronutrients present different functions that support plant defense. Manganese is part of the structure of some secondary metabolites such as phenolic compounds. Nickel is involved in the activity of the antioxidant system and zinc like iron is involved in mechanisms against pathogens. When there is an imbalance with zinc deficiency, plants generate reactive oxygen species (ROS) as a defense against pathogen attack; however, ROS are known to be the reason why growth is inhibited in plants [50,51].

### 7.1. Antioxidants as Plant Defense Systems

Processes such as photosynthesis and respiration, as well as the regulation of ROS production for defense purposes, have been described as essential mechanisms for the adaptation of plants to terrestrial ecosystems, due to the release of oxygen, impacting the evolution of the processes of life on the planet. ROS molecules are highly toxic when they accumulate in cells and may damage DNA, proteins, and lipids. However, ROS have been shown to be essential in growth and development processes, stomatal movement, and plant–microorganism interactions [52].

Plants are extremely rich in compounds with antioxidative activity, and their presence is ubiquitous. Antioxidants are molecules capable of inhibiting or quenching free radical reactions and delaying or preventing cell damage, and, in lower concentrations than the potential substrate which might be oxidized, significantly delay or hinder its oxidation. It has been reported that antioxidants can be classified as water-soluble (ascorbate, glutathione, and phenols), and liposoluble (tocopherols, tocotrienols, and carotenoids) which act as the foremost prominent low relative molecular mass antioxidants [53].

The synthesis and accumulation of phytochemicals in plants depend on several factors, including genetics, environmental factors (microclimate, location, growing season, soil type, and nutrients), post-harvest storage, and processing conditions. Mineral composition, soil type, temperature, light, and water content are among the frequently reported factors that affect the total phytochemical content in plants [54].

### 7.2. Plant Diseases Caused by a Deficit or Excess of Nutrients in Soil

Plants require sunlight, water, and nutrients for the correct development of their metabolism. Nutrients are required to supply the needs of the plant, play a fundamental role in the life cycle, and are involved in plant nutrition. Mineral nutrients are structural/functional constituents of enzymes or can act as activators and regulators [55]. Nutrient deficiency or toxicity in plants is a consequence of their availability and demand and are influenced by their relative mobility. The movement of the necessary elements to the root for plant development depends on the concentration gradient formed by the absorption and utilization of elements, root interception, and mass flow. In addition, the distribution of necessary inorganic elements in the plant is carried out by the xylem and phloem; transport which varies with plant species [56]. Macronutrient deficiency affects plant metabolism and photosynthesis by reducing electron carriers, decreasing CO_2_ capture, and decreasing the efficiency of metabolic pathways. Macronutrient deficiency is related to the production of reactive oxygen species which can affect normal plant development [57].

Nutrient availability is influenced by the decomposition processes of organic matter, fertilizers, soil chemistry, contaminants, leaching, erosion, and weathering of rocks. Soil–plant interactions are influenced by ionic activity in the soil solution [58]. Nutrient toxicity occurs when the level of soluble nutrients in the soil exceeds its tolerance threshold. The osmotic effect due to Na^+^ in soil causes stomatal closure, reduces transpiration, disturbs plant water status, and inhibits leaf expansion, while the specific ion effect reduces the plant’s ability to uptake other ions such as Ca^2+^, K^+^, and Mg^2+^, affecting the distribution of essential nutrients in plants resulting in the premature senescence of leaves, yield reduction, and plant death [59].

There is a dynamic equilibrium between nutrient reserves and the soil solution, which is influenced by the rate of ion replenishment (capacity factor) of the soil and by its ionic activity (intensity). The interaction between capacity and intensity depends on pH and soil structure. The pH affects the availability of micronutrients and can lead to nutrient toxicity. In very acidic soils, the pH generates manganese toxicity, aluminum toxicity, and molybdenum deficiency. In alkaline soils, boron toxicity and deficiency of iron, zinc, and manganese occur [58].

The production of phenolic compounds in *Theobroma cacao* varies depending on the area and variety of cocoa grown as reported by Alvarez et al. [60] who indicated that depending on the cocoa genotype, soluble phenolic compounds and total phenols are higher in humid tropical areas and dry forests compared to semi-humid areas. This variation is associated with the plasticity of cocoa to adapt to the areas where it is are grown. In addition, it is also known that the production of phenols is a response of the plant to water stress [61]. However, more studies are needed to differentiate the type of phenols produced in each case.

Recent studies have reported that nutrient imbalance due to excess fertilizer causes variation in the production of secondary metabolites. In the case of nitrogen, an excess of this nutrient reduces the production of phenolic compounds and flavonoids [62]. However, soil enrichment with nutrients (N, P, and K) as well as an attack by pathogens induces an increase in the activity of glucanase, peroxidase, and chitinase enzymes, leading to an increase in the amount of alkaloids, terpenes, and phenolic compounds, especially phytoalexins that help in the defense of palm plants against pathogen attack or preventing insects from taking it as food [63]. Deficiencies of P, S, and Mg increase phenol concentrations [64].

**Figure 3:**
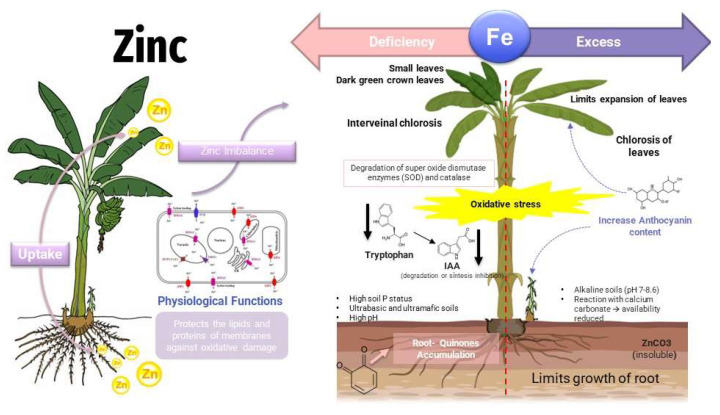
Effects of zinc in plants—deficit/toxicity.

Zn phytotoxicity becomes visible from a foliar concentration above 300 mg/kg, manifesting in yield reduction, growth retardation, reduced export of photo-assimilates from leaves to roots, and chlorosis caused by Fe deficiency due to reduced chlorophyll synthesis and chloroplast degradation (Figure 3) [51].

**Figure 4:**
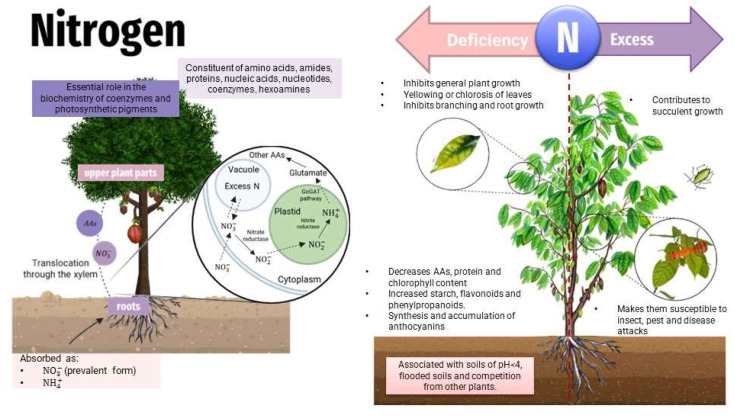
Effects of nitrogen in plants—deficit/toxicity.

Nitrogen excess or deficiency can negatively influence the plant metabolism (Figure 4). An excess of nitrogen produces a darker green coloration on leaves, promoting a heady growth in length, and can also induce succulent growth (in thickness), making plants prone to insect, pest, and disease attacks [50]. Nitrogen-deficient growth media results in decreased contents of photosynthetic pigments (chlorophyll and carotenoids), thereby reducing the photosynthetic performance (particularly CO_2_ assimilation). Nitrogen deficiency or excess increases the production of reactive oxygen species (ROS) in plants, which results in the lipid peroxidation of cell membranes [65].

Nitrogen and phosphorus deficiencies directly affect phenylpropanoid accumulation and lignification [64].

**Figure 5:**
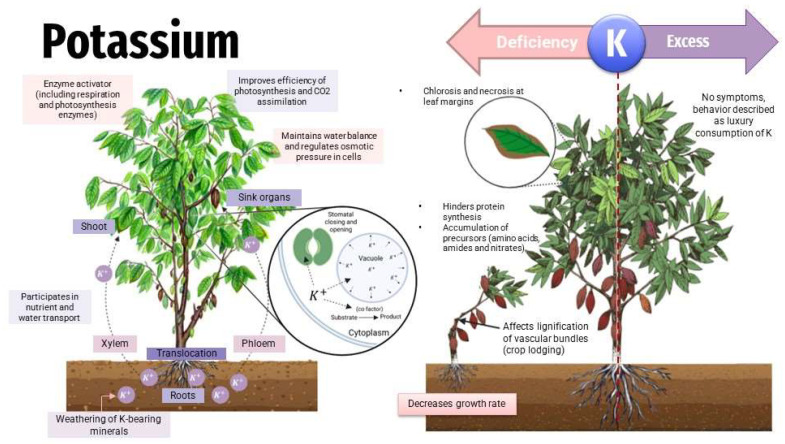
Effects of potassium in plants—deficit/toxicity.

Potassium has a vital role in nitrogen metabolism (Figure 5), ensuring the optimal plant growth [66], playing a role such as the activator of enzymes, is very important for cell growth, stimulates and controls ATPase in the plasma membrane, regulates cell osmotic pressure, and regulates the stomatal opening and closing [67]. A pH 6.5–7.5 assures the most K availability to plants. While an excess of K has no effects on plants, the K deficiency may be observed in the yellowing of the older leaf continuing with necrosis [68].

**Figure 6:**
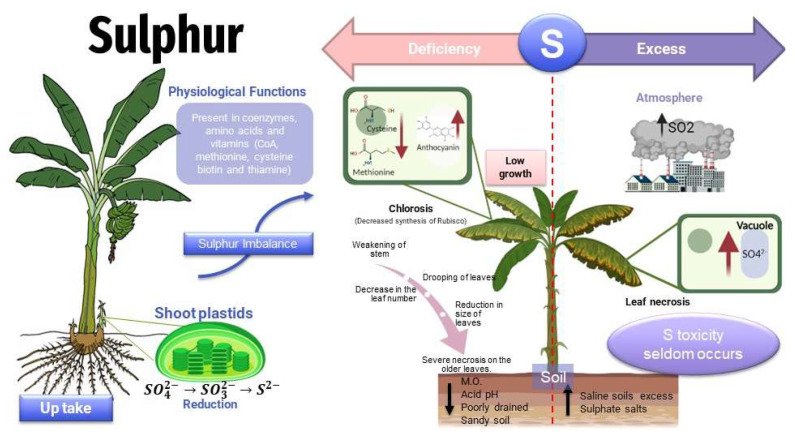
Effects of sulfur in plants—deficit/toxicity.

Sulfur toxicity problems are unusual but can occur in saline soils with excess sulfate salts. As a result of industrial activities and coal burning, atmospheric sulfur can increase in concentration to over 50 ug/m^3^, concentrations that produce foliar necrosis (Figure 6), leading to plant death [50].

**Figure 7:**
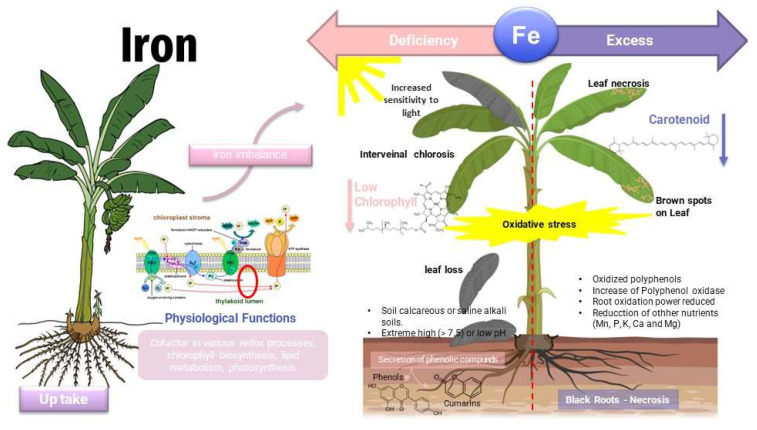
Effects of iron in plants—deficit/toxicity.

Iron toxicity (Figure 7) is considered severe when leaf coloration turns purple–brown. It causes growth retardation, affects the root system by reducing its quantity, and rough and damaged roots of dark brown or black color can be observed [50]. Although Fe is present in sufficient amounts in the soil, under alkaline conditions its bioavailability is limited. When there is a depletion of Fe, chlorophyll and other photosynthetic pigments, such as anthocyanins and carotenoids, decrease as Fe is essential for their biosynthesis [69]. Iron deficiency induces the plants’ ability to down-regulate the gene expression of nitrate reductase and glutamate synthase, accompanied by a greater accumulation of organic acids and flavonoids. In cases of Fe excess, a positive regulation of the genes related to peroxidase against the toxicity of this micronutrient has been reported [70]. The common levels of iron in banana plantations are variable with averages of 404, 367, and 284 ppm at the foliar level in banana farms with intensive production. In the dry season, Fe comes into contact with the air and oxidizes, making nutritional imbalance possible by precipitating in cultivated soils. The iron availability for the banana plant depends on the pH of the soil; in acidic soils it is easily available, while in alkaline or neutral soils Fe is insoluble, causing its deficiency in the plants. Iron excess toxicity in bananas (consisting of the marginal necrosis of old leaves) is rare and can happen in the clayey soils with little oxygen and rainy seasons [71]. Low soil levels of Fe can increase the release of phenolic acids from roots [64].

The synergism or antagonism of elements such as Ca, K, Mg, and Fe are affected by the pH and how it influences the absorption of water and nutrients from the soil. The pH is affected by organic matter and how organic matter increases the Fe content in complex ways. The deficiency of the iron element is observed in calcareous-type soils, with a high Mn content and poor drainage. The rapid oxidation of Fe allows oxides and hydroxides to form in soils with different compositions, degrees of oxidation, and therefore different solubilities that affect assimilation by the plant. The high concentration of iron causes phosphorus retention and consequently a nutritional imbalance in the plant species [71].

Boron mediates the change in the concentration and metabolism of phenolic compounds in vascular plants; its deficiency causes an increase in the concentration of phenols due to the stimulation of the enzyme phenylalanine-ammonium lyase (PAL). On the other hand, it also produces qualitative changes in its phenolic compounds [72]. Boron deficiency in plants causes a series of difficulties in their development due to the role it plays. This element is responsible for stimulating, inhibiting, or stabilizing enzymes, being relevant in the transport of sugars across the membrane, the metabolism of auxins, nitrogen compounds and phenols, as well as the synthesis of lignin and flavonoids. It has been observed that its insufficiency causes a deficit of the pyrimidine base, hindering DNA synthesis, translation, and transcription, as well as the growth and differentiation of plant tissues. Photosynthetic capacity and transport of photosynthetic products is also affected [73]. Boron toxicity occurs in areas with arid and semi-arid soils with 5 ppm of boron, a concentration considered toxic for several types of crops. When there is boron intoxication, plants show yellowing from the tips of the leaves that progresses to premature necrosis and when they die the leaves fall. Another symptom is the appearance of black spots on old leaves [50].

Phytotoxicity caused by boron affects growth parameters with more pronounced effects on shoots than on roots [70]. In banana plants, excess boron is immobilized to a greater extent by the phloem in the leaf margins than in the roots or stems, in order to keep it away from metabolic sites. In addition, some toxicity tests with boron have reported that K, Mn, and Cl concentrations are lower, while Ca and Mg concentrations are higher in the leaves of control plants compared to those treated with B excess [50]. In banana plants, boron in excess is immobilized to a greater extent by the phloem in the leaf margins than in the roots or stems, in order to keep it away from metabolic sites.

**Figure 8:**
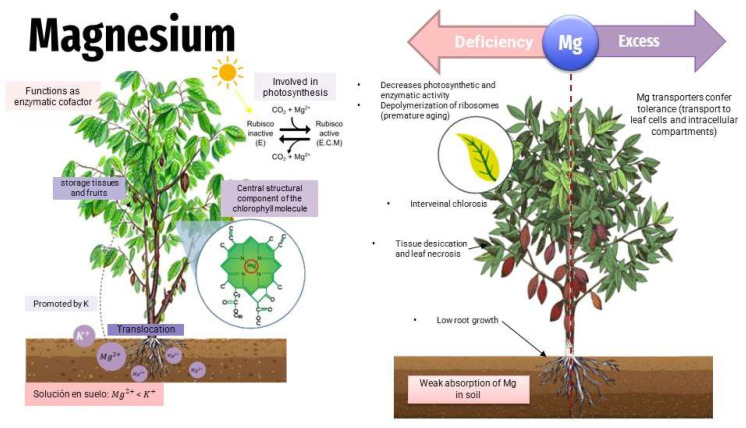
Effects of magnesium in plants—deficit/toxicity.

Magnesium is an essential micronutrient involved in the structure of proteins and photosynthetic enzymes. Mg can cause toxicity in flooded soils, which have a reduced character and also in acid soils with pH < 3. The effects of Mg toxicity on plants are initially observed in older leaves where brown spots occur (Figure 8). In cocoa, Mg toxicity is manifested by yellowish or pale green irregular spots that in this particular case initially affect the young leaves [50]. Its deficiency is observed in dry, calcareous, and sandy soils, reducing crop yields [70].

**Figure 9:**
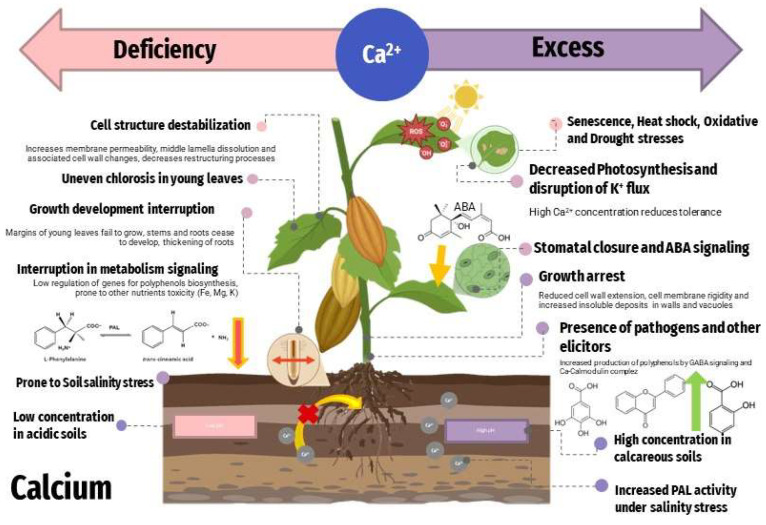
Effects of calcium in plants—deficit/toxicity.

Plants have calcium in an average concentration of 125 µmol/gram of dry weight. Ca^2+^ is generally found in soil but it is relatively insoluble (e.g., CaCO_3_) in its prevalent form. There are some species (e.g., *Trichoderma*) which have the capacity to acidify the surrounding environment by secreting organic acids, in this way solubilizing the phosphates, micronutrients, and mineral cations. From the other side, the simultaneous addition of calcium cations together with biocontrol agents improves the activity of biocontrol agents, that is, through a synergistic act [74]. Calcium enters the plant cells through Ca^2+^—permeable ion channels in their plasma membranes [75]. Calcium is necessary as a component of the cell wall and to neutralize anions. Calcium deficiency can cause a light green color on the uneven chlorosis of young leaves, brown scorching of new leaf tips, poor root growth, and short and thickened roots [76]. The direct role of Ca^2+^ in the synthesis of polyphenolic compounds has been demonstrated and Ca^2+^ supplementation has been shown to increase antioxidant activity in plants [74] (Figure 9).

Copper is one of the essential minerals for plant development. It is related to regulatory proteins, acts as a structural element, and participates in numerous processes of the plants such as photosynthetic electron transport, oxidative stress responses, mitochondrial respiration, and hormonal signaling [77]. Chlorosis is the main manifestation of copper toxicity, being superficially similar to iron deficiency, in addition to inhibiting root growth [50]. Although it is a relevant micronutrient in growth media and important in several biochemical and physiological pathways, its presence in high concentrations makes it toxic to plants. Cu ions have a stimulating effect on the production of secondary metabolites in plants. Normally, copper in soil has been found to induce the synthesis of alkaloids, shikonin synthesis, and betalain and digitalin production. At higher concentrations, Cu^2+^ may indirectly act as a prooxidant [74].

Nickel is higher in excessively humid soils with low humus content and in soils of light granulometry. When soils have a low pH, Ni becomes more accessible to plants. An excess of nickel hinders plant transpiration and decreases moisture content, stomatal conductance, chlorophyll synthesis, and the rate of photosynthesis. Enzymes involved in the Calvin cycle are negatively affected and plant growth is inhibited. Among the symptoms manifested by the plant are chlorosis, yellowish streaks on leaves or white leaves, and necrosis from the edges of the leaves [50].

Chlorine toxicity is usually observed in saline soils, manifested by leaf damage, with burns from the tips or margins, bronzing, leaf abscission, and premature yellowing leading to a low yield and quality of later growth stages [50].

The aspects highlighted by us are consistent with those found by Meya et al., 2020 [78], Mihai et al., 2022 [79], Behera et al., 2021 [80], etc. In the case of banana, Meya et al., 2020 [78] proved that there were significant differences between banana growth from three different altitudinal gradients in volcanic soils; also, fertilization with N in different concentrations showed differences in the studied aspects. In the case of cocoa, Mihai et al., 2022 [79] underlined that there were differences concerning the phytochemical composition and antioxidant and sensory properties of the Arriba variety of cocoa beans originating from different geographical regions of Ecuador depending on nutritional soil status. Behera et al., 2021 [80] indicated the importance of the optimum nutrient concentrations (K, Ca, and Mg) in soils to ensure proper vegetative growth [81].

## 8. Conclusions

Ecuador, due to its edaphic and climatic characteristics, is characterized by a high biodiversity and also is an important source of crops of commercial interest (banana, cocoa, and African palm) highly exported to the rest of the world.

There are many studies which have confirmed that an excess and deficiency of nutrients leads to major problems regarding the culture of the crops.

For better growth and development but also protection (against pathogens) in relation to plant species of economic interest, extensive research on the interaction of nutrients from the soil and plant is needed.

## Figures and Tables

**Table 1:** Ecuadorian soil types and their characteristics.

Soil Orders	Characteristics
Alfisols	very productive for agriculture due to its relatively high native fertility, moderately leached soils typically found under hardwood forested areas or mixed vegetation areas that have average amounts of moisture contain eluviation of aluminum and iron suspended in clay particles, has a “clay skin” [10] develop mainly in areas with steep slopes with a fairly high drainage, or flat areas with poor drainage in temperate regions, tropical or subtropical zones formed under dense deciduous forest vegetation, grasses, meadows
Andisols	developed on pyroclastic materials deposited by volcanic eruptions contain minerals with little crystalline imogolite and allophanes weather rapidly, forming amorphous mixtures of aluminum and silicate in sub-humid and humid regions with a good accumulation of humus high natural productivity medium textures (sandy loam, loam, or silty loam) moderate to weak structure good to moderately excessive drainage black soils on the surface and brown with depth [11] unique and distinctive characteristics due to the representative materials—andic properties: a low bulk density, a highly variable load, and a high phosphate and moisture retention capacity, a variable cation exchange capacity [12]
Aridisols	in arid regions with a climatic regime where evapotranspiration is higher than precipitation during most of the year, with an aridic temperature regime. In arid zones, the physical and chemical reactions of rock alteration occur with less intensity than in humid zones, where temperature and precipitation favor many of these processes contain soluble salts that limit the growth of vegetation physical weathering is the main formative process daily variation > 30 °C between midday and early morning in many cases combined with the lack of water (because it evaporates quickly) produces physical wear on the rocks favoring rocks disintegration. water content is very low to nonexistent for most of the year, leading to limited leaching abundant calcium carbonate making them quite alkaline, and unsuitable for plants that are not adapted to water stress and extreme drought [13]
Entisols	appear in areas of ravines with constant alluvium that do not allow development in depth, erosion, stoniness, excessive thick elements, susceptibility to flooding, and permanent water saturation are its main problems for use potentially very fertile soils due to the different alluviums received that support intensive agriculture [14]
Histosols (peats)	20–30% organic material characterized by low bulk densities (<0.3 g/cm^3^), low bearing capacity, and subsidence when drained [15] reside in swamps, bogs, and marshes where anaerobic conditions and restricted drainage result in a low rate of organic matter decomposition relative to production, and this is the reason for their carbon accumulation, are commonly associated with extremely wet landscapes, extremely acidic soils, nutrient-deficient, andic properties, and permafrost [16]
Inceptisols	young or incipient soils, slightly acid or slightly alkaline pH, high cation exchange capacity, low to medium organic matter [15] in areas with environmental conditions that facilitate the continuous washing of exchangeable bases (high rainfall, high temperature, and steep slopes) their base saturation is ~50%, with black surface horizons and deeper horizons of yellow to reddish color, a product of oxidation [17]
Mollisols	rich in organic matter, characterized by having a superficial horizone (mollic epipedon) and an argillic or cambic horizon in their subsoil. pH ranges from strongly acidic to strongly alkaline [18] develops in a variety of climatic regimes, from dry to very humid or from warm to very cold located in tertiary structural and hilly reliefs of the Coast and the basin floor reliefs with volcanic-sedimentary fillings of the inter-Andean Valley vegetation—grassland, forests develop in places with precipitation ranging between 200 and 800 mm/year used for the cultivation of cocoa, soft corn, hard corn, sugarcane, beans, fruit trees, and potatoes [9]
Oxisols	yellow and reddish coloration found in tropical and subtropical regions composed of a mixture of quartz, free iron oxides, kaolinite, and aluminum [19] characterized by low alterable minerals, low cation exchange capacity, low pH, and relatively high permeability which gives them high resistance to erosion when cultivated [20] high mineral content of iron and aluminum oxides allows stabilizing organic compounds through their surface area and binding sites high temperatures and rainfall cause high microbial decomposition resulting in low soil productivity [21] fertilizers are used to compensate for nutrient deficits, phosphorus being the most restrictive nutrient due to the ability of sesquioxide-rich clays to fix this mineral [19]
Ultisols	strongly leached acid soils, low native fertility found in tropical and humid temperate areas [15] formed by high environmental temperatures, has a thin argillic horizon and a low content of organic matter and bases (Na, K, Ca, Mg) generally below 2.5% with an accumulation of illuvium clay. coloration depends on the degree of Fe hydration, giving reddish brown or reddish tones in its oxidized form and yellowish brown or yellowish in its hydrated form [22,23]

**Table 2:** Characteristics of the edaphic and climatic factors for the ideal development of the crops of commercial interest.

Crops	Edaphic Characteristics	Climatic Factors
Banana	soils with good drainage, relative heterogeneity, with a good water requirement, a high available K content [47]	warm zones, the average annual temperature of 28 °C, with extremes of 18 and 35 °C. water requirement: 150 mm/month average supplementary irrigation is sometimes necessary about 1500 h of light/year below 300 m.a.s.l. [47]
Cocoa	loam to sandy loam texture at a depth of 0.6 m deep, fertile, and drained, rich in organic matter pH should be close to neutral abundant organic matter [42]	the humid tropics 1250 mm annual rainfall average optimum temperature 24–26 °C altitudes below 1300 m.a.s.l. [42]
*African palm*	clay loam to silty clay loam soils, flat or slightly sloping with a depth of 0.60 m pH between slightly acidic (5.6–6.5) and practically neutral (6.5–7.5) well drained with a salinity of less than 2 dS/m^2^ [48]	not less than 200 mm rainfall/month on average average annual temperature 25–28 °C; can grow at 22–33 °C. approximately 1400 h of light/year altitude not higher than 600 m.a.s.l. [48]

**Table 3:** Critical information on nutrients in relation to plants after Karthika et al. [50].

Nutrient	Plant-Usable Form	Average Concentration in Plant Tissue	Biochemical Functions
Nutrients that are part of carbon compounds			
N	$NO_{3}^{-},\;NH_{4}^{+}$	1.50%	Constituent of amino acids, amides, proteins, nucleic acids, nucleotides, coenzymes, hexoamines, etc.
S	$SO_{4}^{2-}$	0.10%	Component of cysteine, cystine, methionine, proteins, lipoic acid, biotin, coenzyme A, adenosine-5′-phosphosulphate, glutathione
Nutrients that are important in energy storage or structural integrity			
P	$H_{2}PO_{4}^{-},\;HPO_{4}^{-}$	0.20%	Component of sugar phosphates, nucleic acids, nucleotides, coenzymes, phospholipids, phytic acid, etc. Has a key role in reactions that involve ATP
B	$H_{3}BO_{3}{}^{-}$ $,\;H_{2}BO_{3}$ $,\;HBO_{3}^{2-}$ $,\;BO_{3}^{3-}$	20 mg/Kg	Complexes with mannitol, mannan, polymannuronic acid; constituents of cell walls; involved in cell elongation, nucleic acid metabolism
Si			Deposited as amorphous silica in cell walls; contributes to cell wall mechanical properties (rigidity and elasticity)
Nutrients that remain in ionic form			
K	$K^{+}$	1.00%	Required as a cofactor for more than 40 enzymes, cation used in establishing cell turgor
Ca	$Ca^{2+}$	0.50%	Constituent of the middle lamella of cell walls; second messenger in metabolic regulation; cofactor by some enzymes involved in ATP hydrolysis and phospholipids
Mg	$Mg^{2+}$	0.20%	Required by many enzymes involved in phosphate transfer. Constituent of the chlorophyll molecule
Cl	$Cl^{-}$	100 mg/Kg	Required for the photosynthetic reactions involved in O_2_ evolution
Mn	$Mn^{2+}$	20 mg/Kg	Required for activity of some dehydrogenases, decarboxylases, kinases, oxidases, and peroxidases. Involved with other cation-activated enzymes and photosynthetic O_2_ evolution
Na			Involved with the regeneration of phosphoenolpyruvate in C4 and CAM plants.Substitutes for potassium in some functions
Nutrients that are involved in redox reactions			
Fe	$Fe^{2+}$	100 mg/Kg	Constituent of cytochromes and non-haem iron proteins involved in photosynthesis, N_2_ fixation, and respiration
Zn	$Zn^{2+}$	20 mg/Kg	Constituent of alcohol dehydrogenase, glutamic dehydrogenase, carbonic anhydrase, etc.
Cu	$Cu^{2+}$	5 mg/Kg	Component of ascorbic acid oxidase, tyrosinase, monoamine oxidase, uricase, cytochrome oxidase, phenolase, laccase, and plastocyanin
Mo	$MoO_{4}^{2-}$	0.1 mg/Kg	Constituent of nitrogenase, nitrate reductase, and xanthine dehydrogenase
Ni	$Ni^{2+}$	0.1 mg/Kg	Constituent of urease. In N_2_-fixing bacteria, constituent of hydrogenases
H	$H_{2}O$	6%	
O	$H_{2}O,\;O_{2}$	45%	
C	$CO_{2}$	45%	

## Data Availability

Not applicable.

## References

[1] Palma-Henríquez J. (2018). Las regiones naturales. Master’s Thesis.

[2] Portilla F. (2018). Introducción. Agroclimatología del Ecuador.

[3] Medina Carranco N.C. (2019). Corgánico de la Economía Social de los Conocimientos, Creatividad e Innovación. Título de Ingeniero Estadístico.

[4] Palacios-Estrada M., Massa-Sánchez P., Martínez-Fernández V.A. (2018). Cambio climático y contaminación ambiental como generadores de crisis alimentaria en la américa andina: Un análisis empírico para Ecuador. Investig. Oper..

[5] Cruzatty L.C.G., Vollmann J.E.S. (2012). Caracterización de suelos a lo largo de un gradiente altitudinal en Ecuador. Rev. Bras. Cienc. Agrar..

[6] Varela L.A., Ron S.R. (2018). Geografía y Clima del Ecuador. BIOWEB. Pontificia Universidad Católica Del Ecuador, Quito, Ecuador. https://bioweb.bio/geografiaClima.html/.

[7] Acaro L., Córdoba A. (2021). Evolution in banana exports and impact of economic development, province of El. Rev. Científica.

[8] Sanchez Mendez J.A. (2016). Suelos Apropiados y Fértiles, para Impulsar el Desarrollo de la Agricultura en la Región Costa del Ecuador. Tenure Thesis.

[9] Calvache-Ulloa A.M. La Fertilidad de los Suelos del Ecuador, Isotopes in Ecuadorian Agriculture. Proceedings of the XII Congreso Latinoamericano Agronomía.

[10] Bekele D., Alemayehu B. (2020). The characteristics, distribution and management of alfisols: A Review. Int. J. Hum. Resour. Manag..

[11] Elizondo-Alvarado M.J. (2015). Suelos De Costa Rica Orden Andisol. Boletín Técnico.

[12] Jaramillo J.D.F. (2009). Variabilidad espacial de las propiedades ándicas de un Andisol hidromórfico del oriente antioqueño (Colombia). Rev. Fac. Nac. Agron. Medellín.

[13] Kissel R.A., Parrish J.T. (2016). Chapter 7: Soils of the Southwestern US. The Teacher-Friendly Guide to the Earth Science of the Southwestern US.

[14] Sigtierras, Sistema Nacional de Información y Gestión de Tierras Rurales e Infraestructura Tecnológica (2017). Memoria explicativa del Mapa de Órdenes de Suelos del Ecuador.

[15] University of Idaho Ultisols. Soil and Water Systems. https://www.uidaho.edu/cals/soil-orders/ultisols.

[16] Aide M.T., Aide C., Braden I., Sarvajayakesavalu S., Charoensudjai P. (2020). Soil Genesis of Histosols and Gelisols with a Emphasis on Soil Processes Supporting Carbon Sequestration. Environmental Issues and Sustainable Development.

[17] Espinosa J., Moreno J., Bernal G. (2018). The Soils of Ecuador.

[18] Hernández Muñoz C.I. (2018). Estimación del Secuestro de Carbono Edáfico en el Ecosistema Herbazal de Páramo del Territorio Hídrico del Río Cebadas, Chimborazo, Ecuador. Bachelor’s Thesis.

[19] Eswaran H., Reich P.F., Hillel D. (2005). World Soil Map. Encyclopedia of Soils in the Environment.

[20] Hartemink A.E., Zhang Y., Bockheim J.G., Curi N., Silva S.H.G., Grauer-Gray J., Lowe D.J., Krasilnikov P., Sparks D.L. (2020). Soil horizon variation: A review. Advances in Agronomy.

[21] Ye C., Bai T., Yang Y., Zhang H., Guo H., Li Z., Li H., Hu S. (2017). Physical access for residue-mineral interactions controls organic carbon retention in an Oxisol soil. Sci. Rep..

[22] Chinchilla M., Mata R., Alvarado A. (2011). Caracterización y clasificación de algunos ultisoles de la región de Los Santos, Talamanca, Costa Rica. Agron. Costarric..

[23] Karim A., Hifnalisa, Jufri Y., Fazlina Y.D., Megawati. (2022). Distribution of organic carbon in Ultisol soils with citronella and pine vegetation, at Gayo Highlands, Aceh. IOP Conf. Ser. Earth Environ. Sci..

[24] Rivera Grunauer R.E. (2019). Características Físicas, Ubicación Geográfica y Calidad del Suelo Agrícola de las Provincias de la Costa Ecuatoriana. Bachelor’s Thesis.

[25] Moreno J., Yerovi F., Herrera M., Yánez D., Espinosa J., Haro R. (2022). Suelos Ecuador—Suelos de la Costa. Suelos del Ecuador: Clasificación, uso y manejo.

[26] Bravo C., Benítez D., Vargas J.C., Reinaldo A., Torres B., Marín H. (2015). Environmental Characterization of Agricultural Production Units in the Ecuadorian Amazon Region, Subjects: Pastaza and Napo. Rev. Amaz. Cienc. Y Tecnol..

[27] Vargas Y., Nicolaide J., Calero A., Vizuete O. (2016). Agroforestería Sostenible Agroforestería Sostenible en la Amazonía Ecuatoriana No 2.

[28] Juca L.C., Aguirre P.U., Vivanco N.A. (2021). Ecuador: Análisis económico del desarrollo del sector agropecuario e industrial en el periodo 2000–2018. Rev. Cient. Tecnol. UPSE.

[29] INEC (2022). Encuesta de Superficie y Producción Continua.

[30] Igwe D.O., Ihearahu O.C., Osano A.A., Acquaah G., Ude G.N. (2021). Genetic Diversity and Population Assessment of *Musa* L. (Musaceae) Employing CDDP Markers. Plant. Mol. Biol. Rep..

[31] Martínez C.C., Cayón S.G., Ligarreto M.G. (2015). Physiological attributes of banana and plantain cultivars of the Colombian Musaceae Collection. Agron. Colomb..

[32] Buitrago-Bitar M.A., Enríquez-Valencia A.L., Londoño-Caicedo J.M., Muñoz-Flórez J.E., Villegas-Estrada B., Santana-Fonseca G.E., Buitrago-Bitar M.A., Enríquez-Valencia A.L., Londoño-Caicedo J.M., Muñoz-Flórez J.E. (2020). Molecular and morphological characterization of *Musa* spp. (Zingiberales: Musaceae) cultivars. Boletín Científico. Cent. Museos. Mus. Hist. Nat..

[33] Daquilema S.E.G., Villa A.I.E. (2018). Factores que influyen en la producción del plátano en el Ecuador, 2014–2016. Cienc. Digit..

[34] Ibarra A. (2020). Analysis of Banana Exports in the Multipartes Commercial Framework Between Ecuador and the European Union. Obs. Econ. Latinoam..

[35] Nair K.P. (2020). Tree Crops. BioScience.

[36] Minimol J.S., Suma B., Shija T.K., Shilpa K.S. (2020). Genotypic and seasonal variations affecting yield attributes of cocoa (*Theobroma cacao* L.) varieties. J. Agrometeorol..

[37] Akeredolu M.I., Laseinde T. Fruiting patterns of cacao as affected by shading regimes. Proceedings of the International Conference on Industrial Engineering and Operations Management.

[38] López A., Segovia D. (2017). Producción y Comercialización de Cacao Fino de Aroma en el Ecuador—Año 2012-2014.

[39] Gobierno Provincial de Imbabura (2020). Contratación del Servicio de Consultoría de la Agenda Productica de la Provincia de Imbabura.

[40] Banco Central del Ecuador (2022). Boletín de Análisis Agropecuario.

[41] Nyombi K. (2019). Diagnosis and management of nutrient constraints in bananas (*Musa* spp.). Fruit Crops: Diagnosis and Management of Nutrient Constraints.

[42] Arvelo G.L.D., Arce S., Delgado López T., Arvelo Sánchez M.Á. (2017). Manual Técnico del Cultivo de Cacao: Prácticas Latinoamericanas.

[43] Carrillo Zenteno M., Cevallos Sandoval V., Cedeño García C., Gualoto Gualoto W., Mite Vivar F., Arturo Navarrete Parraga M., Ortega Cedillo D., Ortega Cedillo J., Quintero Román L., Racines Jaramillo M. (2015). Manual del Cultivo de la Palma Aceitera.

[44] FAO (2022). Soils for Nutrition: State of the Art.

[45] Jones B. (2012). Plant Nutrition and Soil Fertility Manual.

[46] Mohammed S., Mohammed S. (2018). Climatic Conditions for Crop Production. Tomorrow’s Agriculture: NFT Hydroponics-Grow within Your Budget.

[47] Puyutaxi A., Marcelo F. (1992). Clima, Suelos, Nutrición y Fertilización de Cultivos en el Litoral Ecuatoriano.

[48] Ganchozo W., Huaraca H. (2017). Guía Para Facilitar el Aprendizaje en el Manejo Integrado del Cultivo de Palma Aceitera (Elaeis guineensis, Jacq).

[49] Bechtold U., Field B. (2018). Molecular mechanisms controlling plant growth during abiotic stress. J. Exp. Bot..

[50] Karthika K.S., Rashmi I., Parvathi M.S. (2018). Biological Functions, Uptake and Transport of Essential Nutrients in Relation to Plant Growth. Plant Nutrients and Abiotic Stress Tolerance.

[51] Cabot C., Martos S., Llugany M., Gallego B., Tolrà R., Poschenrieder C. (2019). A Role for Zinc in Plant Defense Against Pathogens and Herbivores. Front. Plant Sci..

[52] Ramírez Gómez M., Rodríguez A. (2012). Plant defense mechanisms and responses in the arbuscular mycorrhizal symbiosis: A review. Rev. Colomb. Biotecnol..

[53] Dumanović J., Nepovimova E., Natić M., Kuča K., Jaćević V. (2021). The Significance of Reactive Oxygen Species and Antioxidant Defense System in Plants: A Concise Overview. Front. Plant Sci..

[54] Li H., Tsao R., Deng Z. (2012). Factors affecting the antioxidant potential and health benefits of plant foods. Can. J. Plant Sci..

[55] De Bang T.C., Husted S., Laursen K.H., Persson D.P., Schjoerring J.K. (2021). The molecular–physiological functions of mineral macronutrients and their consequences for deficiency symptoms in plants. New Phytol..

[56] Nazar R., Iqbal N., Masood A., Khan M.I.R., Syeed S., Khan N.A. (2012). Cadmium Toxicity in Plants and Role of Mineral Nutrients in Its Alleviation. Am. J. Plant Sci..

[57] Tewari R.K., Yadav N., Gupta R., Kumar P. (2021). Oxidative Stress Under Macronutrient Deficiency in Plants. J. Soil Sci. Plant Nutr..

[58] Langridge P., Reynolds M.P., Braun H.J. (2022). Micronutrient Toxicity and Deficiency. Wheat Improvement: Food Security in a Changing Climate.

[59] Hussain N., Sohail Y., Shakeel N., Javed M., Bano H., Gul H.S., Zafar Z.U., Frahat Z.H.I., Ghaffar A., Athar H.R. (2022). Role of mineral nutrients, antioxidants, osmotic adjustment and PSII stability in salt tolerance of contrasting wheat genotypes. Sci. Rep..

[60] Alvarez J.F., Quintero D., Rodríguez M., Rea R., Sosa D. (2018). Evaluation of phenolic compounds, lignin, amino acids and carbohydrates in *Theobroma cacao* L. from three different climate regions in Venezuela. Emir. J. Food Agric..

[61] Janani P., Kumar N., Jegadeeswari V. (2019). Effect of water deficit on physiological and biochemical responses in cocoa (*Theobroma cacao* L.) clones. J. Pharmacog. Phytochem..

[62] Hasan H., Manan F.A. (2020). Total Phenolic and Flavonoid Content of *Elaeis guineensis* Leaves Treated with Different Amount of Nitrogen-Potassium Fertilizer. Int. J. Life Sci. Biotechnol..

[63] Sahebi M., Hanafi M., Rafii M., Azizi P., Abiri R., Kalhori N., Atabaki N. (2017). Screening and Expression of a Silicon Transporter Gene (Lsi1) in Wild-Type Indica Rice Cultivars. BioMed Res. Int..

[64] Ramakrishna A., Ravishankar G.A. (2011). Influence of abiotic stress signals on secondary metabolites in plants. Plant Signal. Behav..

[65] Yañez-Mansilla E., Cartes P., Reyes-Díaz M., Ribera A., Rengel Z., Alberdi M. (2014). Leaf nitrogen thresholds ensuring high antioxidant features of *Vaccinium corymbosum* cultivars. J. Soil Sci. Plant Nutri..

[66] White P.J., Karley A.J. (2010). Potassium Cell Biology of Metals and Nutrients.

[67] Xu X., Du X., Wang F., Sha J., Chen Q., Tian G., Zhu Z., Ge S., Jiang Y. (2020). Effects of Potassium Levels on Plant Growth, Accumulation and Distribution of Carbon, and Nitrate Metabolism in Apple Dwarf Rootstock Seedlings. Front. Plant Sci..

[68] Hopkins W.G., Huner N.P.A. (2009). Introduction to Plant Physiology.

[69] Santos C.S., Ozgur R., Uzilday B., Turkan I., Roriz M., Rangel A.O.S.S., Carvalho S.M.P., Vasconcelos M.W. (2019). Understanding the Role of the Antioxidant System and the Tetrapyrrole Cycle in Iron Deficiency Chlorosis. Plants.

[70] Chrysargyris A., Höfte M., Tzortzakis N., Petropoulos S.A., Di Gioia F. (2022). Micronutrients: The Borderline Between Their Beneficial Role and Toxicity in Plants. Front. Plant Sci..

[71] Moran J.F., Becana M., Iturbe-Ormaetxe I., Frechilla S., Klucas R.V., Aparicio-Tejo P. (1994). Drought induces oxidative stress in pea plants. Planta.

[72] Bhatt D.S., Debnath S.C. (2021). Genetic diversity of blueberry genotypes estimated by antioxidant properties and molecular markers. Antioxidants.

[73] Brdar-Jokanović M. (2020). Boron Toxicity and Deficiency in Agricultural Plants. Int. J. Mol. Sci..

[74] Jurić S., Sopko Stracenski K., Król-Kilińska Ż., Žutić I., Uher S.F., Đermić E., Topolovec-Pintarić S., Vinceković M. (2020). The enhancement of plant secondary metabolites content in *Lactuca sativa* L. by encapsulated bioactive agents. Sci. Rep..

[75] White P.J., Broadley M.R. (2003). Calcium in plants. Ann. Bot..

[76] Nathan M. (2011). Diagnosing Nutrient Deficiencies and Toxicities. https://ipm.missouri.edu/MEG/2011/6/Diagnosing-Nutrient-Deficiencies/.

[77] Marschner H. (1995). Mineral Nutrition of Higher Plants.

[78] Meya A., Ndakidemi P.A., Mtei K.M., Swennen R., Merckx R. (2020). Optimizing Soil Fertility Management Strategies to Enhance Banana Production in Volcanic Soils of the Northern Highlands, Tanzania. Agronomy.

[79] Mihai R.A., Landazuri Abarca P.A., Tinizaray Romero B.A., Florescu L.I., Catană R., Kosakyan A. (2022). Abiotic Factors from Different Ecuadorian Regions and Their Contribution to Antioxidant, Metabolomic and Organoleptic Quality of *Theobroma cacao* L. Beans, Variety “Arriba Nacional”. Plants.

[80] Behera J.K., Suresh K., Shukla A.K., Kamireddy M., Mathur R.K., Majumdar K. (2021). Soil and leaf potassium, calcium and magnesium in oil palm (*Elaeis guineensis* Jacq.) plantations grown on three different soils of India: Status, stoichiometry and relations. Ind. Crops Prod..

[81] Breure C.J. (1985). Relevant factors associated with crown expansion in oil palm (*Elaeis guineensis* Jacq.). Euphytica.

